# Characterization of wheat (*Triticum aestivum* L.) accessions using morpho-physiological traits under varying levels of salinity stress at seedling stage

**DOI:** 10.3389/fpls.2022.953670

**Published:** 2022-07-25

**Authors:** Hafiz Ghulam Muhu-Din Ahmed, Yawen Zeng, Humayun Raza, Dur Muhammad, Muhammad Iqbal, Muhammad Uzair, Mueen Alam Khan, Rashid Iqbal, Ayman EL Sabagh

**Affiliations:** ^1^Department of Plant Breeding and Genetics, Faculty of Agriculture and Environment, Islamia University of Bahawalpur, Bahawalpur, Pakistan; ^2^Biotechnology and Germplasm Resources Institute, Yunnan Academy of Agricultural Sciences, Kunming, China; ^3^Department of Botany, Institute of Pure and Applied Biology, Bahauddin Zakariya University, Multan, Pakistan; ^4^National Institute for Genomics and Advanced Biotechnology (NIGAB), National Agricultural Research Centre, Islamabad, Pakistan; ^5^Department of Agronomy, Faculty of Agriculture and Environment, Islamia University of Bahawalpur, Bahawalpur, Pakistan; ^6^Department of Agronomy, Faculty of Agriculture, Kafrelsheikh University, Kafr El Sheikh, Egypt; ^7^Department of Field Crops, Faculty of Agriculture, Siirt University, Siirt, Turkey

**Keywords:** wheat, salinity, stress, PCA, correlation, yield

## Abstract

Abiotic stresses are the major stressors affecting wheat (*Triticum aestivum* L.) production worldwide. The world population is increasing continuously. It is very difficult to feed the population because one-third world’s population consumes wheat as a staple food. Among all abiotic stresses, salinity is one that led to a drastic reduction in wheat crop fitness and productivity. Thus, understanding the effects of salinity stress becomes indispensable for wheat improvement programs which have depended mainly on the genetic variations present in the wheat genome through conventional breeding. Therefore, an experiment was conducted using a complete randomized design with four replications, to determine the selection criteria for salinity-tolerant germplasm based on morphophysiological traits at the seedling stage. Three levels of salt solutions, i.e., 4, 8, and 12 dSm^–1^ were applied and the performance of different genotypes under these three salinities levels was observed. Results depicted that leaf water content and relative water content were correlated with each other. Notably, selection based on these traits increased the performance of other characters. The genotypes G11, G13, G18, G22, and G36 performed best in the salinity stress. So, these genotypes are considered salinity-tolerant genotypes. The genotypes G4, G17, G19, G30, and G38 performed worst in the stress and these were salinity-susceptible genotypes. From the results of the principal component (PC) analysis, the first five PCs were indicated to have a substantial genetic variation from the total of 14 PCs. These PCs showed 75, 73, 65.324, and 65.162% of total variation under normal, salinity level 4, 8, and 12 dSm^–1^, respectively. Stomatal conductance, fresh shoot weight and fresh root weight, and dry shoot weight and dry root weight were not significant and negatively associated with all other traits studied, except for relative water and leaf water content. Overall, the results suggested that selection based on leaf water content and relative water content at the seedling stage would genetically improve salinity tolerance. Genotypes with good performance under salt stress conditions may be useful in future breeding programs and will be effective in developing high-yielding salt-tolerant wheat varieties.

## Introduction

Wheat (*Triticum aestivum* L.) is a widely grown cereal crop in many regions of the world (Cuong et al., 2020; Chang et al., 2022). It is a member of the Poaceae family. It is mostly grown between 30°N and 60°N latitudes, and between 27°S and 40°S latitudes, and up to 3,000 m above sea level (Deng et al., 2005). It can also withstand a broad range of temperatures and humidity levels, with 250–2,000 mm of annual precipitation (Deng et al., 2005). About 20% of calories and 55% of carbohydrates are provided by wheat across the globe. Both growth and yield of wheat are negatively influenced by salinity (Arzani and Ashraf, 2017). Wheat bread has high vitamins B, thiamine, and B2-riboflavin content (Rakaščan et al., 2019). Wheat is used in the manufacturing of leavened bread, pasta, flat and steamed breads, cakes, cookies, noodles, and couscous. The global wheat production capacity in the year 2020–2021 was 768.9 million metric tonnes (Lark et al., 2021). Pakistan is the world’s sixth-largest wheat grower, producing 26 million metric tonnes per year. It contributes to 9.2% of agricultural value-added and 1.8% of gross domestic product (GDP). Wheat crop output reached high up to 8.1% over the previous year’s output (Rana, 2020). There are several biotic and abiotic stresses which cause a substantial decrease in plant production (Aslam et al., 2013; Muhammad et al., 2021). In the last past few years, global warming and climate change have severely affected agricultural crop yield (Skirycz and Inzé, 2010; Ibrar et al., 2021). Among various abiotic stresses, soil salinity is a major obstacle to wheat production in many parts of the world, leading to yield losses (El Sabagh et al., 2020). Salinity impairs the seedling establishment, stunts plant growth, causes poor reproductive development, and ultimately declines the crop yield (Dhakal et al., 2021).

Globally, 20% of agricultural land is salinized, although the area and intensity of salinity are increasing (Biabani et al., 2013). Globally, increasing salinity levels is a critical concern and a major constraint to food production (Alom et al., 2016). In Pakistan, about 14% of irrigated land has been reduced by salinity, while 64% of yield losses are due to salinity (Akram et al., 2007). Over 2.5 million hectares of irrigated land are afflicted by severe surface salinity, namely, 3% in Punjab, 18% in Sindh, and 2% in the Khyber Pakhtunkhwa (KPK). The moderately affected areas are 4% in Punjab, 10% in Sindh, and 2% in the Khyber Pakhtunkhwa (KPK). According to estimation, 4.5 Mha out of 79.61 Mha area of Pakistan is salt-affected. Due to the combined effects of high osmotic potential and specific ion toxicity, excessive soil salinity can greatly impair seed germination and seedling development. Salinity also alters the ultrastructural cell components, disturbs the photosynthesis machinery, damages the membranous structure, increases the reactive oxygen species production, and reduces the enzymatic activity, which limit the growth and yield of crops. The most vulnerable stages to salt stress are the seed germination stage and early seedling development (Muhammad and Hussain, 2010). Salinity affects around 6% of the world’s total land, with 20% of arable land, and 33% of irrigated land being particularly vulnerable (Safdar et al., 2019; Yu et al., 2019).

Furthermore, land salinization is increasing, and almost 10 million hectares (ha) of agricultural land are lost each year due to salt buildup caused by human activities and other climate change-related variables (Smajgl et al., 2015; Isayenkov, 2019). Studies revealed that salinity stress at the germination stage is most important for the screening of many species (Ghoulam et al., 2002). Many scientists noticed that salinity stress during germination in wheat cultivars showed a great variation (Kandil et al., 2012). The growth of the wheat plant is reduced due to salinity stress but the response is varied from genotype to genotype. Different growth stages have different impacts on salt tolerance (Flowers et al., 1997). In seedling experiments, many scientists reported that the wheat crop has three main stages of establishment: germination, emergence, and early seedling growth, which are particularly sensitive to salinity (Saboora et al., 2006). Plant growth and productivity are dramatically reduced by salinity stress, which can result in a major reduction in crop output (Tao et al., 2021).

Photosynthesis is the most important physiological process for plant life, and it is mainly affected by the environment. Several seedling characteristics like shoot length, root length, root fresh weight, and shoot fresh weight are affected by salt stress (Asif et al., 2020). Because roots are in the soil and absorb water, therefore root and shoot lengths are among the most influential criteria for salt stress evaluation. The salinity decreases the germination rate and consequently plant density is decreased (Biabani et al., 2013). In general, chlorophyll content decreased in response to salinity stress (Ouhaddach et al., 2018). The selection of salt-tolerant cultivars at the seedling stage is seen to be a good technique. As a result, the current study was carried out to investigate seed germination responses and selection criteria for the breeding procedure based on germination, seedling, and physiological characters of wheat of 40 different genotypes under different levels of salinity.

## Materials and methods

### Experimental location and plant material

A total of 40 genotypes with different genetic backgrounds were provided by the Regional Agriculture Research Institute Bahawalpur, Pakistan. Their seeds were stored in plastic bags at room temperature 25°C. The experiment was conducted at the wirehouse of the Department of Plant Breeding and Genetics, Islamia University of Bahawalpur, Pakistan. During the year 2021, 40 wheat genotypes were sown using a complete randomized design with four treatments. Genotypes used in this study are given in Supplementary Table 1.

### Treatments and traits evaluation

Out of four treatments, one treatment is normal (N) and three treatments were stressed. In the stressed treatments, three levels of salt solution were applied: 4 (ST1), 8 (ST2), and 12 dSm^–1^ (ST3). The data of the following traits like shoot and root lengths were measured using a meter scale, shoot and root fresh weights were recorded through electric balance, chlorophyll content index through spade meter, and stomatal conductance was measured using a leaf photometer. The weight of seedling roots and shoots were taken in milligrams after oven drying at 70°C for 72 h. The data of final germination percentage (Ellis and Roberts, 1981; Islam and Karim, 2010), germination index (Karim et al., 1992), seedling height reduction (Ruan et al., 2002), relative dry weight (Nazeer et al., 2020), root shoot ratio (Nazeer et al., 2020), relative water content (Nazeer et al., 2020), and leaf water content (Huang et al., 2019) were also measured in the current study.

### Statistical analysis

Analysis of variance given by Steel and Torrie (1996) was used to analyze the mean differences between salinity levels and genotypes, and their interaction, at 0.05 and 0.01 significant levels. Pearson’s correlation coefficient was assessed to find the linear relation between various physiological and morphological attributes. The differentiated genotypes were selected based on Principal component analysis (PCA) and radar analysis (Ogunbayo et al., 2005). For this purpose, XLSTAT software was used. The statistically significant PCs were selected using eigenvalues standards as established by Kaiser (1960). The radar analysis was constructed by using Microsoft Excel.

## Results

**FIGURE 1:**
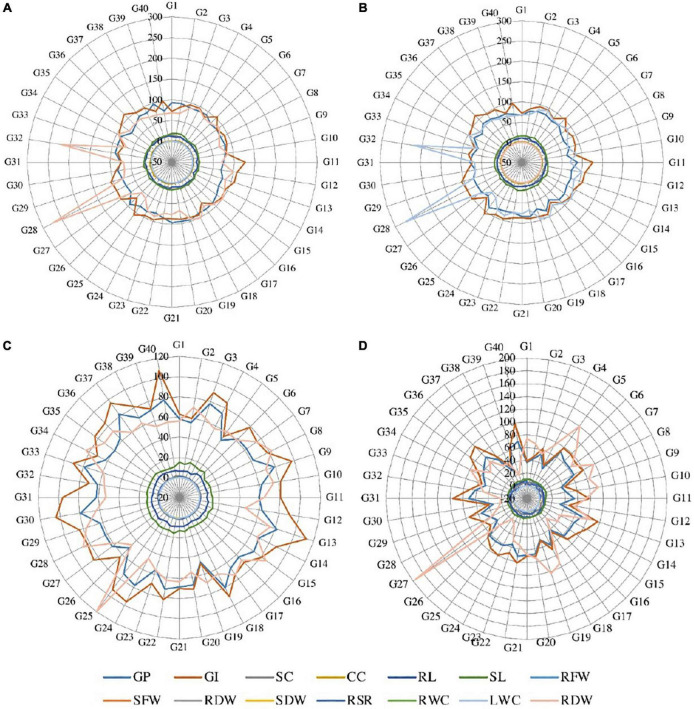
Radar graph between the studied traits under **(A)** normal condition, **(B)** salinity stress at 4 dSm^–1^, **(C)** salinity stress at 8 dSm^–1^, and **(D)** salinity stress at 12 dSm^–1^. GP, germination percentage; GI, germination index; SC, stomatal conductance; CC, chlorophyll content; RL, root length; SL, shoot length; RFW, root fresh weight; SFW, shoot fresh weight; RDW, root dry weight; SDW, shoot dry weight; RWC, relative water content; LWC, leaf water content; RSR, root shoot ratio; RDW, relative dry weight.

Analysis of variance for germination percentage showed that highly significant variations were present among the genotypes under salinity conditions (Table 1). The genotypes were classified using a radar graph (Figure 1) based on studied parameters across three salinity levels and one normal level. The results in Table 2 showed that two genotypes (G11 and G20) attained a higher value for germination percentage under salinity stress level 4 dSm^–1^ (87.78 and 85.00%, respectively). The genotype (G13) showed the highest germination percentage (81.94) under salinity stress level 8 dSm^–1^. The genotype (G36) attained the highest germination percentage (71.67%) under salinity level 12 dSm^–1^. Therefore, the genotype G36 had the maximum germination percentage under salinity stress 12 dSm^–1^ which could be considered a salinity-tolerant genotype. While G38 has the lowest germination percentage that can be considered as salinity-susceptible genotypes under all levels. Genotypes G11, G13, and G40 achieved the highest germination index under salinity stress levels 4, 8, and 12 dSm^–1^, respectively. The genotype G40 had the maximum germination index (98.33) showing that the genotype is salinity tolerant and G38 has the lowest germination index showing that the genotype is salinity susceptible. Meanwhile, the genotypes G30 (12.30), G40 (11.70), and G27 (6.87) have the maximum stomatal conductance under salinity stress levels 4, 8, and 12 dSm^–1^. G27 (6.87) revealed maximum stomatal conductance under 12 dSm^–1^ salinity stress, which is considered salinity tolerant and G36 has the minimum stomatal conductance, which is considered a salinity-susceptible genotype according to Table 2.

**TABLE 1:** Analysis of variance (ANOVA) mean squares of 40 genotypes at the seedling stage under normal and salinity stress conditions.

	Replication	Treatment	Error1	Genotypes	Interaction	Error	Total
			R*T		T*G	R*T*G	
SOV/df	2	3	6	39	117	312	479
GP	18,730.90	18,128**	271.1	319.9**	241.7**	110.4	
SC	149.08	3,592.42**	159.44	3.88**	5.36**	1.67	
CC	0.991	104.693**	1.368	0.540**	0.636**	0.124	
GI	1,149.70	17,328.5**	622.5	1,259.5**	364.5*^ns^*	377.6	
RL	3.114	530.539*	79.365	4.727*^ns^*	5.671**	4.021	
SL	6.152	944.743**	7.7	9.062**	5.579**	4.586	
RDW	0.00124	0.00734**	0.00052	0.00059*^ns^*	0.00077*^ns^*	0.00069	
SDW	0.00275	0.02357**	0.00148	0.00117*^ns^*	0.00144**	0.00111	
RFW	0.055	0.00278*	0.03171	0.00256**	0.00263*^ns^*	0.00283	
SFW	0.0134	0.35216**	0.00825	0.00571*^ns^*	0.00756**	0.00436	
RWC	0.1148	0.39943**	0.10329	0.03002*^ns^*	0.02953*^ns^*	0.02683	
LWC	0.36514	0.71688**	0.22665	0.04992*^ns^*	0.05736**	0.05375	
RSR	8.55833	2.58529**	5.21292	0.71139**	0.66081*^ns^*	0.56705	
Relative DW	1,372.40	23,284.3**	3,714	5,615.9**	2,483.3*^ns^*	2,790.50	

**Highly significant (0.01); *significant (0.05). ns, non-significant; GP, germination percentage; GI, germination index; SC, stomatal conductance; CC, chlorophyll content; RL, root length; SL, shoot length; RFW, root fresh weight; SFW, shoot fresh weight; RDW, root dry weight; SDW, shoot dry weight; RWC, relative water content; LWC, leaf water content; RSR, root shoot ratio; Relative DW, relative dry weight.

**TABLE 2:** Performance of wheat genotypes under salinity stress.

Trait		Best-performing genotypes mean values (salinity-tolerant)	Worst-performing genotypes: mean values (salinity-susceptible)
GP	N	G19 and G39 (96.67) followed by G21 (95.83) and G5 (91.11)	G12 (70.00) followed by G11 (73.61) and G31 (74.00)
	ST1	G11 (87.78) followed by G20 (85.00) and G18 (83.33)	G33 (364.17) followed by G30 (66.57) and G1 (66.67)
	ST2	G13 (81.94) followed by G18 (80.0) and G33 (79.17)	G19 (47.50) followed by G26 (50.83) and G2 (55.00)
	ST3	G36 (71.67) followed by G40 (70.83) and G32 (62.50)	G38 (30.00) followed by G4 (33.33) and G1 (36.67)
GI	N	G11 (125.55) followed by G36 (113.33) and G 12 (108.33)	G17 and G26 (71.29) followed by G31 (71.75) and G1 (71.76)
	ST1	G11 (125.55) followed by G36 (113.33) and G12 (108.33)	G26 (71.29) followed by G31 (71.75) and G1 (71.76)
	ST2	G13 (112.50) followed by G30 (104.17) and G31 (95.83)	G19 (48.98) followed by G5 (55.35) and G2 (59.35)
	ST3	G40 (98.33) followed by G13 (96.67) and G31 (95.60)	G38 (36.67) followed by G4 (38.52) and G1 (38.89)
SC	N	G37 (17.07) followed by G38 (17.30) and G1 (16.10)	G6 (10.60) followed by G7 (10.93) and G4 (11.03)
	ST1	G30 (12.30) followed by G9 (12.17) and G8 (12.00)	G37 (6.80) followed by G38 (6.90) and G3 (6.97)
	ST2	G40 (11.70) followed by G32 (11.62) and G20 (11.55)	G36 (4.52) followed by G5 (5.65) and G39 (5.70)
	ST3	G27 (6.87) followed by G3 (6.54) and G6 (6.47)	G31 (3.41) followed by G13 (3.57) and G25 (3.59)
CC	N	G27 (5.13) followed by G30 (3.74) and G23 (3.31)	G17 (1.33) followed by G1 (1.36) and G15 (1.39)
	ST1	G39 (2.27) followed by G21 (2.23) and G23 (2.22)	G13 (0.73) followed by G6 (0.75) and G8 (0.77)
	ST2	G6 (0.39) followed by G35 (0.23) and G24 (0.20)	G32 (0.02) followed by G21 (0.03) and G8 (0.04)
	ST3	G1 (1.17) followed by G6 (0.74) and G5 (0.60)	G39 (0.10) followed by G37 (0.12) and G27 (0.14)
RL	N	G15 (16.60) followed by G22 (15.87) and G35 (15.00)	G14 (7.23) followed by G11 (8.23) and G5 (10.93)
	ST1	G11 (13.07) followed by G3 (12.77) and G16 (12.63)	G37 (7.77) followed by G33 (8.07) and G21 (8.40)
	ST2	G6 (9.83) followed by G5 (9.60) and G23 (9.47)	G32 (6.07) followed by G4 (6.23) and G18 (6.37)
	ST3	G2 (10.38) followed by G1 (10.07) and G33 (9.77)	G15 (2.89) followed by G38 (5.57) and G40 (6.73)
SL	N	G2 (20.10) followed by G3 (19.60) and G30 (19.37)	G14 (13.40) followed by G29 (13.70) and G5 (14.07)
	ST1	G22 (20.00) followed by G4 (19.10) and G21 (18.80)	G10 (12.37) followed by G9 (12.97) and G35 (13.47)
	ST2	G3 (16.27) followed by G6 (15.70) and G25 (14.67)	G38 (10.40) followed by G40 (10.67) and G7 (10.83)
	ST3	G9 (11.63) followed by G25 (11.23) and G22 (10.93)	G4 (9.10) followed by G14 (9.13) and G12 (9.27)
RFW	N	G29 (0.137) followed by G38 (0.129) and G39 (0.118)	G13 (0.034) followed by G 16 (0.040) and G36 (0.041)
	ST1	G28 (0.32) followed by G40 (0.21) and G27 (0.13)	G37 (0.04) followed by G36 (0.05) and G7 (0.06)
	ST2	G25 (0.091) followed by G24 (0.089) and G22 (0.087)	G16 (0.42) followed by G3 (0.043) and G11 (0.046)
	ST3	G7 (0.117) followed by G6 (0.106) and G11 (0.091)	G39 (0.061) followed by G38 (0.062) and G37 (0.063)
SFW	N	G40 (0.464) followed by G39 (0.445) and G27 (0.383)	G1 (0.124) followed by G6 (0.149) and G13 (0.163)
	ST1	G19 (0.258) followed by G30 (0.253) and G2 (0.252)	G4 (0.121) followed by G27 (0.152) and G25 (0.154)
	ST2	G3 (0.27) followed by G8 (0.26) and G4 (0.25)	G1 (0.16) followed by G20 (0.17) and G30 (0.18)
	ST3	G2 (0.172) followed by G1 (0.171) and G8 (0.148)	G17 (0.0119) followed by G23 (0.122) and G21 (0.123)
RDW	N	G8 (0.049) followed by G9 (0.048) and G22 (0.047)	G25 (0.019) followed by G15 (0.030) and G14 (0.031)
	ST1	G39 (0.037) followed by G28 (0.035) and G3 (0.034)	G1 (0.020) followed by G2 (0.022) and G5 (0.024)
	ST2	G19 (0.03) followed by G24 (0.029) and G28 (0.028)	G1 (0.01) followed by G10 (0.020) and G35 (0.021)
	ST3	G19 (0.068) followed by G10 (0.067) and G5 (0.065)	G31 (0.005) followed by G39 (0.008) and G37 (0.010)
SDW	N	G 26 (0.082) followed by G25 (0.079) and G35 (0.078)	G28 (0.044) followed by G15 (0.048) and G1 (0.05)
	ST1	G32 (0.202) followed by G28 (0.17) and G16 (0.067)	G12 (0.032) followed by G1 (0.033) and G38 (0.035)
	ST2	G25 (0.151) followed by G8 (0.058) and G35 (0.050)	G12 (0.026) followed by G1 (0.031) and G33 (0.032)
	ST3	G8 (0.049) followed by G6 (0.047) and G5 0.046 ()	G39 (0.014) followed by G40 (0.016) and G29 (0.017)
RSR	N	G25 (3.18) followed by G12 (0.86) and G15 (0.84)	G26 (0.51) followed by G33 (0.54) and G31 (0.55)
	ST1	G12 (2.13) followed by G24 (1.11) and G38 (0.94)	G32 (0.28) followed by G28 (0.34) and G21 (0.38)
	ST2	G12 (0.90) followed by G20 (0.86) and G11 (0.79)	G25 (0.24) followed by G21 (0.38) and G15 (0.40)
	ST3	G32 (8.18) followed by G1 (7.50) and G5 (6.92)	G27 (0.02) followed by G13 (0.03) and G28 (0.07)
LWC	N	G40 (0.42) followed by G39 (0.40) and G29 (0.34)	G20 (−0.06) followed by G31 (−0.09) and G24 (−0.12)
	ST1	G11 (0.31) followed by G40 (0.20) and G15 (0.11)	G19 (−0.01) followed by G22 (−0.02) and G29 (−0.04)
	ST2	G22 (0.18) followed by G4 (0.15) and G3 (0.13)	G34 (−0.01) followed by G30 (−0.02) and G35 (−0.04)
	ST3	G39 (0.10) followed by G31 (0.09) and G30 (0.07)	G3 (−0.01) followed by G17 (−0.04) and G33 (−0.09)
RWC	N	G39 (0.39) followed by G40 (0.37) and G29 (0.29)	G12 (−0.05) followed by G6 (−0.08) and G1 (0.01)
	ST1	G40 (0.203) followed by G2 (0.16) and G15 (0.15)	G39 (−0.012) followed by G8 (−0.014) and G3 (−0.031)
	ST2	G22 (0.204) followed by G3 (0.16) and G9 (0.15)	G34 (−0.02) followed by G25 (−0.32) and G21 (0.02)
	ST3	G39 (0.12) followed by G31 (0.11) and G29 (0.08)	G17 (−0.01) followed by G23 (−0.02) and G2 (−0.03)
Relative DW	N	G8 (272.55) followed by G32 (219.44) and G15 (101.17)	G30 (66.41) followed by G40 (67.04) and G31 (067.61)
	ST1	G28 (272.55) followed by G32 (219.44) and G18 (100.78)	G30 (66.41) followed by G31 (67.61) and G20 (68.07)
	ST2	G25 (119.76) followed by G28 (87.20) and G15 (85.86)	G12 (46.08) followed by G26 (46.34) and G24 (47.62)
	ST3	G27 (194.30) followed by G5 (120.54) and G19 (102.38)	G39 (18.34) followed by G25 (19.40) and G31 (20.18)

N, normal; ST1, salinity stress at 4 dSm^–1^; ST2, salinity stress at 8 dSm^–1^; ST3, salinity stress at 12 dSm^–1^; GP, germination percentage; GI, germination index (%); SC, stomatal conductance; CC, chlorophyll content index; RL, root length (cm); SL, shoot length (cm); RFW, root fresh weight (mg); SFW, shoot fresh weight (mg); RDW, root dry weight (mg); SDW, shoot dry weight (mg); RWC, relative water content; LWC, Leaf water content; RSR, root shoot ratio; Relative DW, relative dry weight.

According to Table 2, G39 has the maximum chlorophyll content (2.27) under salinity stress 4 dSm^–1^ and is considered salinity tolerant and G32 has the lowest chlorophyll content (0.02) under salinity stress 8 dSm^–1^ and is considered salinity tolerant.

Two genotypes (G11 and G3) had the highest root length (13.07 and 12.77 cm) under the salinity stress at the 4 dSm^–1^ level. The genotype G2 attained the highest root length (10.38 cm) under the salinity stress level 12 dSm^–1^, and this genotype is considered salinity-tolerant and the worst-performing genotypes were considered as salinity susceptible. The genotype G32 has the lowest root length (6.07 cm) while the genotype G22 showed the maximum shoot length under salinity stress level and that genotype can be considered a salinity-tolerant genotype. While G4 has the lowest shoot length (9.10 cm), which is considered a salinity-susceptible genotype.

According to our findings, maximum root fresh weight was reported in G28 (0.32 mg) under the salinity stress level 4 dSm^–1^, which is considered a salinity stress-tolerant genotype, and minimum root fresh weight was recorded in G37 under salinity stress level 4 dSm^–1^, which is considered a salinity-stress-susceptible genotype. Similarly, G19 had the maximum shoot fresh weight under salinity stress level 4 dSm^–1^ and that genotype can be considered salinity tolerant. While G25 had the lowest shoot fresh weight (0.15 mg), which is considered a salinity-susceptible genotype. The extremely substantial variation among the genotypes was shown by an ANOVA for shoot fresh weight under normal and studied the salinity stress levels as mentioned in Table 1. According to Table 2, G19 has the maximum root dry weight (0.068 mg) under salinity stress 12 dSm^–1^ and is considered salinity tolerant and G1 has the minimum root dry weight (0.01 mg) under salinity stress 8 dSm^–1^, which is considered salinity susceptible.

According to our results, maximum shoot dry weight was reported in G32 (0.202 mg) under the salinity stress level 4 dSm^–1^, which is considered the salinity stress tolerant, and minimum shoot dry weight was recorded in G39 (0.014 mg) under salinity stress level 12 dSm^–1^, which is considered salinity stress susceptible. The results in Table 2 showed that the two genotypes G32 and G1 attained a higher value for root shoot ratio under salinity stress level 12 dSm^–1^ (8.18 and 7.50). The genotype G32 had the highest root shoot ratio under salinity stress level 12 dSm^–1^ (8.18), which can be considered a salinity-tolerant genotype. While the genotype G27 (0.02) has the lowest root shoot ratio that can be considered a salinity-susceptible genotype.

Therefore, G11 has the maximum leaf water content (0.31) under salinity stress 4 dSm^–1^, which can be considered a salinity-tolerant genotype. While G19 has the lowest leaf water content (−0.01) that can be considered a salinity-susceptible genotype. Consequently, G22 had the maximum relative water content (0.204) under salinity stress 8 dSm^–1^, which can be considered a salinity-tolerant genotype. While G17 had the lowest relative water content (−0.01) that can be considered a salinity-susceptible genotype. Meanwhile, G28 has the maximum relative dry weight (272.55) under salinity stress 4 dSm^–1^, which can be considered a salinity-tolerant genotype. While G39 has the lowest relative dry weight (18.34) that can be considered a salinity-susceptible genotype. ANOVA for root dry weight showed highly significant variations among the genotypes under the salinity stress according to Table 1.

Results of the association of seedling indices in normal and stress conditions were found in this study, which might aid in the development of advanced techniques for the selection of necessary varieties with desired features. The correlation of studied traits under normal and stressful environments is presented in Table 3. The coefficient of correlation of germination index was negative and highly significant with germination percentage under normal environment while positive and highly significant under salinity stress levels 4, 8, and 12 dSm^–1^. Stomatal conductance is an integral trait of leaf in seeding experiments to calculate the transpiration rate. However, the association between the shoot fresh weight and chlorophyll content was highly significant under the normal and the salinity stress level 12 dSm^–1^. The correlation between root shoot ratio and germination percentage was negatively significant under the salinity stress level 8 dSm^–1^, also the association between the root shoot ratio and root dry weight was highly significant under the normal environment and salinity stress levels 4, 8, and 12 dSm^–1^ as mentioned in Table 3. Association between the root length and germination index was negatively significant under normal and salinity stress level 12 dSm^–1^. According to Table 3, the correlation between root length and stomatal conductance was negatively significant under salinity stress level 8 dSm^–1^.

**TABLE 3:** Correlation matrix among wheat seedling traits under normal and stress environment.

Traits	LEVEL	GP	GI	SC	CC	RL	SL	RFW	SFW	RDW	SDW	RSR	RWC	LWC	RDW
GI	N	−0.61**													
	ST1	0.57**													
	ST2	0.81**													
	ST3	0.91**													
SC	N	0.1*^ns^*	−0.11*^ns^*												
	ST1	−0.24*	−0.2*^ns^*												
	ST2	0.11*^ns^*	0.1*^ns^*												
	ST3	−0.42*	−0.41*												
CC	N	−0.25*	0.07*^ns^*	−0.08*^ns^*											
	ST1	0.21*	−0.12*^ns^*	0.07*^ns^*											
	ST2	0.06*^ns^*	0.19*	−0.04*^ns^*											
	ST3	−0.12*^ns^*	−0.1*^ns^*	0.05*^ns^*											
RL	N	0.07*^ns^*	−0.27*	0.11*^ns^*	0.22*										
	ST1	0.04*^ns^*	−0.03*^ns^*	−0.05*^ns^*	−0.17*										
	ST2	−0.25*	−0.08*^ns^*	−0.20*	0.14*										
	ST3	−0.05*^ns^*	−0.25*	0.06*^ns^*	0.37*										
SL	N	0.28*	−0.25*	0.09*^ns^*	0.27*	0.04*^ns^*									
	ST1	0.17*	−0.14*	−0.12*^ns^*	0.05*^ns^*	−0.04*^ns^*									
	ST2	0.02*^ns^*	0.02*^ns^*	0.11*^ns^*	0.11*^ns^*	0.14*									
	ST3	0.1*^ns^*	0.001*^ns^*	−0.04*^ns^*	−0.09*^ns^*	−0.04*^ns^*									
RFW	N	0.1*^ns^*	−0.26*	0.16*	0.22*	0.30*	−0.08*^ns^*								
	ST1	−0.08*^ns^*	0.16*	0.1*^ns^*	−0.14*	−0.16*	−0.22*								
	ST2	0.14*	−0.01*^ns^*	0.15*	0.20*	0.14*	−0.01*^ns^*								
	ST3	0.03*^ns^*	0.04*^ns^*	0.18*	0.38*	0.12*^ns^*	0.08*^ns^*								
SFW	N	0.06*^ns^*	0.01*^ns^*	−0.05*^ns^*	0.48**	0.18*	0.05*^ns^*	0.54**							
	ST1	0.17*	0.32*	−0.02*^ns^*	−0.14*	0.47**	−0.01*^ns^*	−0.06*^ns^*							
	ST2	0.213*^ns^*	−0.02*^ns^*	−0.19*	−0.30*	0.01*^ns^*	0.06*^ns^*	−0.22*							
	ST3	−0.19*	−0.21*	0.15*	0.52**	0.29*	0.05	0.31*							
RDW	N	−0.05*^ns^*	0.21*	0.08*^ns^*	−0.05*^ns^*	0.12*^ns^*	−0.22*^ns^*	0.04*^ns^*	0.1*^ns^*						
	ST1	0.23*^ns^*	0.24*	−0.14*	−0.03*^ns^*	−0.21*	−0.19*	−0.08*^ns^*	−0.16*						
	ST2	−0.16*	−0.15*	−0.29*	−0.19*	−0.05*^ns^*	−0.27*	−0.19*	0.26*						
	ST3	−0.07*^ns^*	−0.15*	0.13*^ns^*	0.35*	0.20*	0.08*^ns^*	0.25*	0.19*						
SDW	N	0.08*^ns^*	−0.19*	0.18*	0.11*^ns^*	0.05*^ns^*	−0.13*^ns^*	0.12*^ns^*	0.11*^ns^*	0.22*					
	ST1	0.001*^ns^*	−0.01*^ns^*	0.12*^ns^*	0.04*^ns^*	−0.02*^ns^*	−0.18*	0.43*	−0.02*^ns^*	−0.12*^ns^*					
	ST2	0.11*^ns^*	0.11*^ns^*	0.02*^ns^*	0.04*^ns^*	−0.02*^ns^*	0.08*^ns^*	0.33*	−0.27*	−0.19*					
	ST3	−0.15*	−0.09*^ns^*	0.35*	−0.13*^ns^*	−0.12*^ns^*	−0.19*	0.16*	0.14*	−0.04*^ns^*					
RSR	N	−0.08*^ns^*	0.26*	0.02*^ns^*	–0.06	0.09*^ns^*	−0.20*	0.20*	0.08*^ns^*	0.98**	0.02*^ns^*				
	ST1	−0.09*^ns^*	0.16*	−0.09*^ns^*	−0.02*^ns^*	−0.20*	−0.09*^ns^*	−0.15*	−0.14*	0.91**	−0.36*				
	ST2	−0.15*	−0.11*^ns^*	−0.06*^ns^*	−0.13*^ns^*	−0.08*^ns^*	0.23*	−0.23*	0.16*	0.54**	−0.60**				
	ST3	0.34**	−0.19*	−0.03*^ns^*	0.36*	0.65**	−0.06*^ns^*	0.13*^ns^*	0.27*	0.55**	–0.13				
RWC	N	0.09*^ns^*	−0.03*^ns^*	−0.06*^ns^*	0.35*	0.26*	0.07*^ns^*	0.70**	0.90**	0.01*^ns^*	−0.08*^ns^*	0.01*^ns^*			
	ST1	0.01*^ns^*	0.21*	−0.05*^ns^*	−0.17*	0.15*	0.08*^ns^*	0.22*	0.45*	−0.25*	−0.60**	−0.08*^ns^*			
	ST2	0.02*^ns^*	−0.06*^ns^*	−0.04*^ns^*	−0.04*^ns^*	0.06*^ns^*	0.13*^ns^*	−0.21*	0.57**	0.001**	−0.84**	0.42*			
	ST3	0.12*^ns^*	0.1*^ns^*	−0.32*^ns^*	0.09*^ns^*	0.09*^ns^*	0.1*^ns^*	−0.13*^ns^*	−0.07*^ns^*	−0.49*	−0.81**	−0.12*^ns^*			
LWC	N	0.09*^ns^*	−0.09*^ns^*	−0.01*^ns^*	0.47*	0.25*	0.1*^ns^*	0.70**	0.94**	0.08*^ns^*	−0.02*^ns^*	0.09*^ns^*	0.92**		
	ST1	0.001*^ns^*	0.11*^ns^*	−0.01*^ns^*	−0.13*^ns^*	0.26*	0.13*^ns^*	0.02*^ns^*	0.62**	−0.31*	−0.61**	0.88**	–0.12		
	ST2	−0.04*^ns^*	−0.1*^ns^*	−0.06*^ns^*	−0.12*^ns^*	0.06*^ns^*	−0.01*^ns^*	−0.11*^ns^*	0.68**	0.16*	−0.84**	0.46*	0.93**		
	ST3	0.15*	0.15*	−0.29*	0.08*^ns^*	0.11*^ns^*	0.11*^ns^*	0.001*^ns^*	0.06*^ns^*	−0.60*	−0.68**	−0.15*	0.94**		
Relative DW	N	−0.12*^ns^*	0.08*^ns^*	−0.15*	−0.02*^ns^*	−0.09*^ns^*	−0.02*^ns^*	−0.1*^ns^*	0.14*	−0.14*	−0.37*	−0.06*^ns^*	0.12*^ns^*	0.15*	
	ST1	−0.02*^ns^*	0.08*^ns^*	0.05*^ns^*	−0.09*^ns^*	−0.07*^ns^*	0.27*	0.58*	−0.05*^ns^*	0.05*^ns^*	0.93**	−0.49*	−0.20*	−0.59*	
	ST2	0.05*^ns^*	0.06*^ns^*	−0.15*	−0.06*^ns^*	−0.1*^ns^*	0.01*^ns^*	0.15*	−0.06*^ns^*	0.23*	0.79**	−0.47*	−0.71**	−0.64**	
	ST3	−0.16*	−0.16*	0.37*	0.16*	0.06*^ns^*	−0.15*	0.27*	0.24*	0.59*	0.76**	0.26*	−0.94**	−0.89**	

**Highly significant (0.01); *significant (0.05); ns, non-significant. N, normal; ST1, salinity stress at 4 dSm^–1^; ST2, salinity stress at 8 dSm^–1^; ST3, salinity stress at 12 dSm^–1^; GP, germination percentage; GI, germination index (%); SC, stomatal conductance; CC, chlorophyll content; RL, root length; SL, shoot length; RFW, root fresh weight; SFW, shoot fresh weight; RDW, root dry weight; SDW, shoot dry weight; RWC, relative water content; LWC, leaf water content; RSR, root shoot ratio; RDW, relative dry weight.

Correlation between leaf water content and shoot fresh weight was highly significant under the normal condition and salinity stress levels 4 and 8 dSm^–1^. Correlation between stomatal conductance and root dry weight was highly significantly correlated under the normal and salinity stress level 12 dSm^–1^ condition while negative and significantly correlated under salinity stress levels 4 and 8 dSm^–1^. Under the salinity stress 4 dSm^–1^, the root dry weight and chlorophyll content were positively associated with root shoot ratio, shoot length, germination index, and germination percentage, and also with root length, shoot fresh weight, leaf water contents, and relative water contents. Meanwhile, root dry weight and chlorophyll content had a positive association with root shoot ratio, shoot length, germination index, germination percentage, root length, shoot fresh weight, leaf water contents, and relative water contents and were negatively correlated with stomatal conductance, root fresh weight, shoot dry weight, and root dry weight. Otherwise, under the salinity stress level (8 dSm^–1^) the root length, shoot fresh weight, root shoot ratio, and relative dry weight is positively associated with leaf water content, relative water content, and shoot length, and also with root fresh weight, chlorophyll content, stomatal conductance, germination index, and germination percentage. Meanwhile also these positively associated traits were negatively correlated with shoot dry weight. However, under the salinity stress level 12 dSm^–1^, the root length, chlorophyll content, root shoot ratio, shoot fresh weight, root fresh weight, and relative dry weight are positively correlated with relative water content, leaf water content, and shoot length, and are also correlated with rood dry weight and shoot dry weight. The germination percentage and germination index were negatively associated with relative water contents, leaf water content, and shoot length, and also with stomatal conductance, root fresh weight, root dry weight, root shoot ratio, root length, and chlorophyll contents.

**FIGURE 2:**
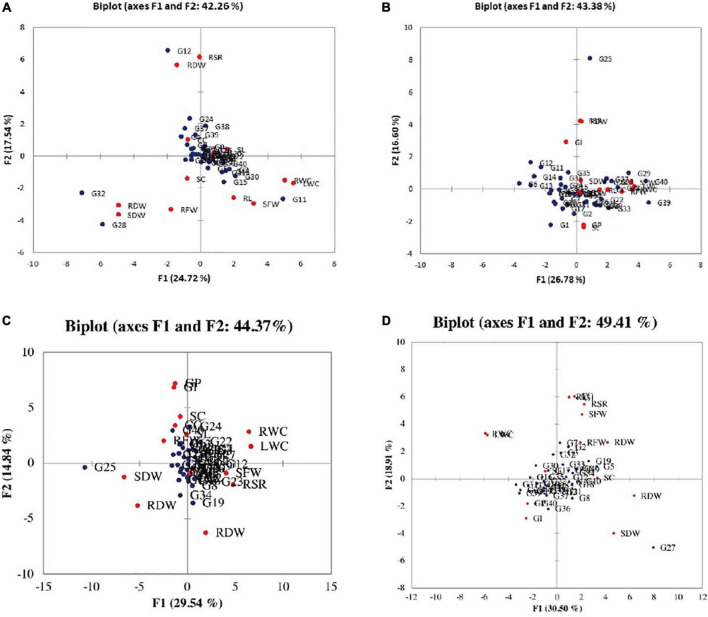
Biplot between the genotypes and the studied traits under **(A)** normal condition, **(B)** salinity stress at 4 dSm^–1^, **(C)** salinity stress at 8 dSm^–1^, and **(D)** salinity stress at 12 dSm^–1^. The red circle shows traits and the blue circle shows genotypes. GP, germination percentage; GI, germination index; SC, stomatal conductance; CC, chlorophyll content; RL, root length; SL, shoot length; RFW, root fresh weight; SFW, shoot fresh weight; RDW, root dry weight; SDW, shoot dry weight; RWC, relative water content; LWC, leaf water content; RSR, root shoot ratio; RDW, relative dry weight.

According to the PCA for parental selection in breeding programs, biplot analysis (Figure 2) was used. Out of 14 principal components of eigenvalues (Table 4), the first five PCs found with eigenvalues larger than 1 under normal salinity conditions were selected. The other nine PCs data were considered non-significant and were not useable for further analysis due to eigenvalues less than 1. The first five PCs showed 75% of the total variation under normal condition. While under salinity level 1 the first five PCs showed 73% of the total variation. Meanwhile, under salinity level 2, the first five PCs showed 65.324% of the total variation, also under salinity level 3, the first five PCs showed 65.162% of the total variation. The 1st PCs marked for 43.38% of the variance under normal condition, 25% of the variance under the salinity stress level 1, 29.5% of the variance under salinity stress level 2, and 30.4% of the variance under salinity level 3 (Table 4).

**TABLE 4:** Eigenvalues, variability, and cumulative of wheat seedling traits under normal and stress environments.

	Level	PC1	PC2	PC3	PC4	PC5	PC6	PC7	PC8	PC9	PC10	PC11	PC12	PC13	PC14
Eigenvalue	N	3.749	2.324	1.974	1.282	1.119	0.936	0.895	0.700	0.432	0.322	0.195	0.053	0.016	0.002
	ST1	3.461	2.456	1.962	1.589	1.230	1.022	0.743	0.671	0.472	0.225	0.081	0.051	0.024	0.013
	ST2	4.135	2.077	1.695	1.309	1.189	0.986	0.870	0.708	0.534	0.239	0.135	0.075	0.046	0.001
	ST3	4.269	2.648	1.964	1.311	1.103	0.779	0.708	0.526	0.369	0.217	0.053	0.028	0.022	0.002
Variability (%)	N	26.780	16.602	14.100	9.156	7.991	6.684	6.393	5.001	3.085	2.301	1.394	0.378	0.115	0.017
	ST1	24.719	17.542	14.017	11.352	5.583	7.300	5.311	4.793	3.372	1.606	0.581	0.361	0.169	0.094
	ST2	20.536	14.837	12.111	9.347	8.493	7.045	6.212	5.054	3.818	1.710	0.967	0.534	0.330	0.008
	ST3	15.495	18.913	14.026	9.364	7.881	5.566	5.057	3.759	2.633	1.546	0.381	0.203	0.158	0.017
Cumulative%	N	43.382	39.382	57.483	66.639	74.630	81.314	87.708	92.708	95.793	98.094	99.489	99.867	99.983	100.000
	ST1	24.719	42.261	56.278	67.630	76.412	83.713	89.023	93.816	97.188	98.795	99.376	99.737	99.906	100.000
	ST2	29.536	44.373	56.483	65.830	74.323	81.368	87.579	92.634	96.451	98.161	99.128	99.662	99.992	100.000
	ST3	30.495	49.408	63.434	72.798	80.680	86.246	91.303	95.062	97.695	99.241	99.622	99.825	99.983	100.000

According to the PCA results, the germination index had a positive association with shoot dry weight, relative dry weight, root shoot ratio, leaf water content, and shoot fresh weight. While root dry weight, root to shoot ratio, shoot dry weight leaf water content, relative water content, and relative dry weight were positively correlated with each other, and also stomatal conductance, root dry weight, root length, chlorophyll contents, root fresh weight, shoot length, and germination percentage were positively correlated with germination index, root dry weight, root to shoot ratio, shoot dry weight, leaf water contents, and shoot fresh weight under normal environment.

## Discussion

Over the years many scientists evaluated different wheat genotypes based on physiological and morphological characteristics. These characteristics were successful in distinguishing the salt-tolerant from the salt-sensitive genotypes under salinity stress conditions, and they detected extremely significant variances among the examined genotypes (Houshmand et al., 2005; El-Hendawy et al., 2009; Shafi et al., 2010; Hasan et al., 2015; Oyiga et al., 2016). Excessive salt concentrations in the root zone obstruct the control of critical ion net-absorption and any imbalance causes a drop in leaf chlorophyll content and photosynthetic efficiency. Excessive salinity causes a reduction in stomatal conductance, showing that it responds to the osmotic stress caused by the salt outside the roots. In a previous study, a reduction in stomatal conductance declined the CO_2_ assimilation rate. The highest averages of these characters were recorded with control treatment (Figure 1). Likewise the rate of germination of wheat genotypes was highly inclined by salinity stress (Alom et al., 2016). It was also reported that the final germination percentage is decreased with an increasing concentration of NaCl in all genotypes (Akbarimoghaddam et al., 2011). The main reason for seed germination failure was the lack of seed water up taking due to a high concentration of salinity (Atak et al., 2006). According to Table 2, those genotypes G11, G13, G18, G22, and G36 which performed well under the salinity stress: 4, 8, and 12 dSm^–1^ were considered salinity-stress-tolerant cultivars and these genotypes could be utilized for a breeding program in future; on the other hand, those genotypes which have low performance were considered as salinity-susceptible genotypes, similar results were reported by Royo and Abió (2003). It is also stated that increasing salinity decreased the seedling vigor and germination index (Fercha and Gherroucha, 2014).

Many researchers examined that the root and shoot length showed a highly significant difference among genotypes at different salinity levels by increasing the salinity level, the length of root and shoot was decreased (Husain et al., 2004; Ibrahim et al., 2016). Increasing salinity levels from 4 to 12 dSm^–1^ NaCl gradually decreased shoot length. The desirable effect of the highest salinity level on root length may be due to damage to the membrane as an important role in the cellular toxicity of NaCl. It could be concluded that increasing salinity levels from 4 to 12 dSm^–1^ NaCl significantly decreased root length, similar results were reported by Fercha and Gherroucha (2014), Esau (1953), and Cutter (1971). The RWC was decreased under salinity stress in studied genotypes and the same result was reported by Akhtar et al. (1998). Increasing the salt content from 4 to 12 dSm^1^ NaCl gradually reduced the fresh weight of the shoots. Lowered water availability for plants owing to the reduced osmotic potential at the root surface, and particular ion toxicity and nutritional imbalance, might explain the lower root fresh weight under saline circumstances. Almost all growth-related parameters, such as root fresh and dry weights, were affected under saline conditions. Increasing salinity level from 4 to 12 dSm^–1^ NaCl gradually significantly decreased root fresh weight, a similar result was observed by Khan et al. (2009). In wheat genotypes, germination% and rate, root and shoot length, and root and shoot dry weight declined as soil salinity increased (Soltani et al., 2004). It was concluded that the shoot fresh weight was significantly affected by the increasing levels of salinity but varied depending on genotypes and the levels of salinity.

Dry weight will provide an exact measurement of biomass reducing fluctuations caused by water content. As a result, germination was proportional to the amount of water absorbed, and germination delay was proportional to the medium’s salt content (Rahman et al., 2008). Seed germination is reduced due to two main reasons: (i) increasing saline levels, viability is lost; (ii) delaying seed germination at salinities that induce some stress but not 100% germination, as some researchers claim (Gulzar et al., 2001). Many scientists reported that in the wheat crop the morphological characteristics were affected at all growth stages in saline stress conditions, which consist of the leaf (size, shape, area, senescence tolerance of cuticle, and waxiness), the root (root hairs, root area, root length, root fresh dry weight, and density), and vegetative parts (height of the plant, diameter, and fresh and dry biomass) (Zheng et al., 2008).

Correlation provides a key concept of association between different traits that contribute to yield, which is beneficial for plant breeders when selecting cultivars with desired attributes. The association between the stomatal conductance and germination percentage under the salinity stress levels 4 and 12 dSm^–1^ was negative and significant. The relationship between stomatal conductance and germination index was negatively significant under the salinity stress level 12 dSm^–1^, a similar result was reported by Khan et al. (2019). Chlorophyll is one of the most important traits for the plants to survive because the plants used chlorophyll for the photosynthetic function to produce their food. According to the agricultural point of view, chlorophyll is directly linked to the overall production of the crop (Živčák et al., 2014). Root length is one of the important traits for the normal growth of a plant because the root hairs absorb the water from the soil and transfer it to the plant *via* xylem tissue but under salinity stress, the salt is accumulated on the surface of the root and the length of root decrease, due to that roots are unable to absorb the water, only salt-resistant germplasm or cultivars survives under the salinity stress (Maas and Nieman, 1978). According to the findings, correspondence between root length and germination percentage was non-significant with each other under normal condition, a similar result was reported by Khan et al. (2010).

Shoot length is an important parameter for breeders for phenotypic selection (Eid, 2009). Shoot length was significantly associated with germination percentage under normal condition and salinity stress level 4 dSm^–1^ and non-significant under salinity stress levels 8 and 12 dSm^–1^, a similar result was reported by Zafar et al. (2019). Meanwhile, the correlation between shoot length and germination index is negatively significant under a normal environment and salinity stress level 4 dSm^–1^ and non-significant under salinity stress levels 8 and 12 dSm^–1^. Fresh root weight refers to the total amount of root growth of a plant. The root mass of a plant needs nutrients, space, and ventilation to grow. The correlation between root fresh weight and root length was significant under normal and salinity stress level 8 dSm^–1^, and negatively significant under the salinity stress level 4 dSm^–1^, similar results were reported by Sabagh et al. (2021). The association between shoot fresh weight and germination percentage was significant under the salinity stress level 4 dSm^–1^. shoot fresh weight is the parameter used to assess overall plant biomass production, which includes leaves and branches (Frick et al., 2011). The association between the root fresh weight and shoot fresh weight was highly significant under the normal and salinity stress level 4 dSm^–1^, a similar result was reported by Kaur et al. (2022). The root system is critical for nutrient and water absorption, and the higher the absorption at the root level, the larger the biomass. Greater root density and root interception for nutrient absorption were associated with a higher root-to-shoot ratio (Nie et al., 2013).

The association between the leaf water content and germination index was negatively significant under salinity stress level 12 dSm^–1^. However, the association between the leaf water content and root length was significant under the normal and salinity level 4 dSm^–1^, a similar result was concluded by Rajabi Dehnavi et al. (2020). However, the relationship between root dry weight and leaf water content was significant under normal conditions and highly significant at salinity stress levels 4, 8, and 12 dSm^–1^. PCA is a multivariate statistical analysis used to examine and simplify large and complex data sets. Furthermore, biplot analysis can be used to select variables, which can be categorized into major groups and subgroups based on homogeneity and dissimilarity. Salinity-tolerant plants employ several physiological and biochemical mechanisms to adapt under salinity stress, there is a lack of robust salinity-tolerant wheat cultivars globally. Therefore, plant physiologists, breeders, and agronomists need to develop an integrated and sustainable strategy to enhance salt tolerance in wheat.

## Conclusion

A total of 40 wheat genotypes were screened out against salinity stress under a complete randomized design. The ANOVA showed a significant variation among the genotypes. According to the PCA, the first five PCs showed a clear difference between genotypes, under the normal and different salinity stress levels (Figure 2). According to the stress levels, the relative water content and leaf water content were positively correlated with each other, and also found in association with other studied traits. The genotypes G11, G13, G18, G22, and G36 performed well under the salinity stress, these genotypes were considered more tolerant to salinity stress and five genotypes were considered salinity stress susceptible (G4, G17, G19, G30, and G38). The best-performing genotypes can be utilized in future wheat breeding programs for developing salinity-stress-tolerant cultivars. However, an integrated approach involving physiological strategies, and biochemical and molecular tools need to be developed to ameliorate salinity effects and boost wheat production on a sustainable basis.

## Data availability statement

The original contributions presented in this study are included in the article/Supplementary Material, further inquiries can be directed to the corresponding authors.

## Author contributions

HA conceived to the idea and conducted the research. HA and HR were carried out investigation. MU, DM, MI, and RI undertook the data analysis. YZ and AE provided the technical expertise to streamline the findings and literature review. HA, HR, MK, and AE helped in writing – original draft. All authors carefully read, revise, and approved the article for submission.
