# Supplementary material for: Characterization of wheat (Triticum aestivum L.) accessions using morpho-physiological traits under varying levels of salinity stress at seedling stage
Source: Front Plant Sci. 2022 Jul 25;13:953670. doi: 10.3389/fpls.2022.953670 (PMC9358580; doi:10.3389/fpls.2022.953670)
Supplement: Supplementary file 1 [file Table_1.DOCX]

Table S1 : Studied wheat genotypes

| Sr. No | Code | Name | Pedigree |
| --- | --- | --- | --- |
| 1 | G1 | Watan | LU26/HD 2179 |
| 2 | G2 | AARI-2011 | SH-88/90A-204//MH97 |
| 3 | G3 | Kohinoor-83 | ORE F1 158/FDL//MFN/2*TIBA63/3/COC |
| 4 | G4 | Chakwal-86 | FORLANI/ACC//ANA or Fln/ACS//ANA |
| 5 | G5 | Bakhtawar-94 | Mentana/Mayo//4-11 |
| 6 | G6 | Bahawal-97 | PFAU'S'/SERI |
| 7 | G7 | Chakwal-50 | ATTILA/3/HUI/CARC//CHEN/CHTO/4/ATTILA |
| 8 | G8 | AS-2002 | KHP/D31708//CMH74A370/3/ENO79/4/R26043/*4NAC |
| 9 | G9 | Bakhtawar-93 | AU/UP301//GLL/SX/3/PEW/4/MAI/MAYA//PEW |
| 10 | G10 | Pasban-90 | INIA F66/TH.DISTICHUM//INIAF66/3/GENARO T81 or INIA F66/A.DISTCHUM//INIA66/3/GEN |
| 11 | G11 | AUqab-2000 | CROW'S'/NAC//BOW'S' |
| 12 | G12 | Iqbal-2000 | BURGUS/SORT 12-13//KAL/BB/3/PAK 81 |
| 13 | G13 | Bakhar-2002 | P102/PIMA//F371/TTR/BOW/3/PVN |
| 14 | G14 | Shafaq-2006 | LU 26/HD 2179/ 2*INQALAB 91 |
| 15 | G15 | Fakhar-e-Sarhad | NORD-DESPREZ(ND)/VG-9144//KALYANSONA/BLUEBIRD/3/YACO/4/VEERY-5 |
| 16 | G16 | Fareed-2006 | PT'S'/3/TOB/LFN//BB/4/BB/HD-832-5//ON/5/G-V/ALD'S'//HPO |
| 17 | G17 | FSD-2008 | PBW65/2*Pastor |
| 18 | G18 | Aas-2011 | PRL/PASTOR//2236 |
| 19 | G19 | FSD-85 | MAYA/MON//KVZ/TRM |
| 20 | G20 | GA 2002 | DWL5023/SNB//SNB |
| 21 | G21 | Galaxy-2013 | Pb96/Watan/MH-97 |
| 22 | G22 | Punjab-85 | KVZ/TRM//PTM/ANA |
| 23 | G23 | Hashim-2008 | JUP/ALD'S'//KLT'S'/3/VEE'S'/6/BEZ//TOB/8156/4/ON/3/6*TH/KF//6*LEE/KF/--------- |
| 24 | G24 | Inq-91 | WL 711/CROW "S" |
| 25 | G25 | Millat-2011 | CHENAB2000/INQ-91 |
| 26 | G26 | Khyber-87 | KVZ/TRM//PTM/ANA |
| 27 | G27 | Kohistan-97 | V-1562//CHRC'S'/HORK/3/KUFRA-I/4/CARP'S'/BJY'S' |
| 28 | G28 | BARS-2009 | PFAU/SERI//BOW |
| 29 | G29 | Kohsar-95 | PSN/BOW |
| 30 | G30 | Lasani-2008 | LUAN/KOH-97 |
| 31 | G31 | Ufaq-2002 | V.84133/V83150 |
| 32 | G32 | Marvi-2000 | CMH-77A917/PKV 1600//RL6010/6*SKA |
| 33 | G33 | Maxi-Pak 65 | PJ/GB55 |
| 34 | G34 | Mehran-89 | KVZ/BUHO//KAL/BB |
| 35 | G35 | FSD-83 | FURY//KAL/BB |
| 36 | G36 | FAKHR-E-SARHAD | PFAU'S'/SERI//BOW'S' |
| 37 | G37 | Moomal-2002 | BUC or BUCS/4/TZPP/IRN46 |
| 38 | G38 | Pak-81 | VEERY. |
| 39 | G39 | Parwaz-94 | V.5648/PARULA or V.5648/PRL |
| 40 | G40 | Chakwal-97 | BUC'S'/FCT'S' |
